# Comparative performances of DNA barcoding across insect orders

**DOI:** 10.1186/1471-2105-11-206

**Published:** 2010-04-27

**Authors:** Massimiliano Virgilio, Thierry Backeljau, Bruno Nevado, Marc De Meyer

**Affiliations:** 1 Royal Museum for Central Africa, Leuvensesteenweg 13, 3080, Tervuren, Belgium; 2 Royal Belgian Institute of Natural Sciences, Vautierstraat 29, 1000, Brussels, Belgium; 3 Department of Biology, University of Antwerp, Groenenborgerlaan 171, B-2020 Antwerp, Belgium

## Abstract

**Background:**

Previous studies on insect DNA barcoding provide contradictory results and suggest not consistent performances across orders. This work aims at providing a general evaluation of insect DNA barcoding and "mini-barcoding" by performing simulations on a large database of 15,948 DNA barcodes. We compared the proportions of correctly identified queries across a) six insect orders (Coleoptera, Diptera, Hemiptera, Hymenoptera, Lepidoptera and Orthoptera), b) four identification criteria (Best Match: BM; Best Close Match: BCM; All Species Barcodes: ASB; tree-based identification: NJT), and c) reference databases with different taxon coverage (100, 500, 1,000, 1,500 and 1,995 insect species).

**Results:**

Analysis of variance revealed highly significant differences among ID criteria and insect orders. *A posteriori *comparisons of means showed that NJT had always a significantly lower identification success (NJT = 0.656, S.D. = 0.118) compared to both BM and BCM (BM = 0.948, S.D. = 0.026; BCM = 0.946, S.D. = 0.031). NJT showed significant variations among orders, with the highest proportion of correctly identified queries in Hymenoptera and Orthoptera and the lowest in Diptera. Conversely, the proportions of correct matches of BM and BCM were consistent across orders but a progressive increase in false identification was observed when larger reference databases were used.

**Conclusions:**

Regardless the relatively low proportion of Type I errors (misidentification of queries which are represented in the reference database) of BM and BCM, the lack of reference DNA barcodes for 98% of the known insect species implies that insect DNA barcoding is heavily biased by Type II errors (misidentification of queries without conspecifics in the database). The detrimental effects of Type II errors could be circumvented if insect DNA barcoding is used to verify the lack of correspondence between a query and a list of properly referenced target species (*e.g*. insect pests). This "negative identification" would only be subjected to Type I errors and could be profitably adopted in insect quarantine procedures.

## Background

DNA barcoding aims at identifying organisms by assessing their degree of DNA sequence similarity to a set of reference taxa. The standard sequence used for this purpose is the mitochondrial COI gene fragment amplified by the "universal primers" of Folmer *et al*. [1]. Sequence similarities are then interpreted using numerical methods such as hierarchical clustering of genetic distances and statistical evaluations of genetic distance thresholds [2]. Recently, DNA barcoding has been explicitly defined as the molecular identification of a species based on the reference sequence with the lowest genetic distance [3]. Yet, other numerical methods have been proposed to improve this approach [4-7].

DNA barcoding is generally considered as a reliable, cost-effective and easy molecular identification tool with a wide applicability across metazoan taxa [8-12]. As such it could be very useful to routinely identify difficult taxa of economic and medical importance. This particularly holds for many insect taxa that comprise large numbers of notorious pest species or disease vectors, whose identification often requires highly specialised taxonomic skills. In addition, DNA barcoding could be pivotal for the identification of certain life stages (*e.g*. eggs, larvae, nymphs or pupae), which are often impossible to identify otherwise. However, despite these highly positive claims, DNA barcoding also seems to suffer from a number of potential limitations when used for the identification of insects [4]. The recent speciation, the prevalence of paraphyly and the regular interspecific hybridisation in many insect taxa, as well as their often poorly-established taxonomy and their high degree of infection by endosymbiotic bacteria such as Wolbachia [11,13-15] may all negatively affect the performance of insect DNA barcoding. Even more importantly, the reliability of insect DNA barcoding may be questioned because insects include >1,000,000 described species and probably millions of still undescribed taxa [15]. This exuberant species richness may, indeed, severely constrain the ability of the DNA barcode reference databases to adequately represent the overwhelming insect taxonomic diversity.

Not surprisingly, insect DNA barcoding has hitherto produced contradictory results. Several studies showed that it is a reliable tool for the molecular identification of Lepidoptera [8,12,16], Hymenoptera [11,17], Coleoptera [18] and Diptera species [19,20]. Yet, other studies questioned the adequacy of DNA barcoding in Lepidoptera [21] and Orthoptera [22], while Meier *et al*. [4] reported a remarkably low identification success for Diptera (<70% in simulations based on >400 taxa). The limited success of DNA barcoding evidenced by Meier *et al*. [4] was attributed to the use of GenBank sequences, which supposedly include a high proportion of misidentified sequences [23]. A closer look at the data of Meier *et al*. [4] shows that 322 out of 449 species in their dataset (corresponding to approximately 72% of taxa and 24% of sequences) are represented by single DNA barcodes. When these DNA barcodes are used as queries in simulations, they necessarily generate incorrect identifications, because there are no other conspecific reference sequences in the dataset with which they can match [24]. Meier *et al*. [4] included species represented by single barcodes in order to better reflect real-life situations, where it is not possible to know in advance if a query has a conspecific match in the database. However, this approach does not allow distinguishing the negative effects of poor taxon coverage from other potential constraints on insect DNA barcoding.

An additional problem with insect DNA barcoding is that the reference databases strongly rely on DNA sequencing of museum material, the success of which is often limited [25,26]. The DNA quality of museum specimens is generally low as it rapidly degrades over time. For example, less than 50% of moth and wasp museum specimens that were fixed for <8 years still yielded useful DNA barcodes [26]. The use of more efficient DNA extraction and repair protocols, and the amplification of multiple overlapping sections of the barcode region, generally improves the DNA barcoding success rates, though at a substantially higher cost [27]. Therefore, several studies proposed to use shorter barcode fragments (mini-barcodes) for molecular identification. These sequences often represent the only molecular information available for an insect species and are considered as temporary proxies for the complete DNA barcode. For these reasons, it would be important to quantify which is the proportion of correct identification guaranteed by mini-barcodes of different lengths and verify if different regions of the barcode fragment are equally informative in the molecular identification of insects.

The Barcoding of Life Initiative is the main consortium (CBOL) coordinating the collection of DNA barcodes and building a worldwide reference database for the molecular identification of species (BOLD, http://www.barcodinglife.org). Currently (June 2009) the BOLD system includes >400,000 insect DNA barcodes. The validation and/or identification of many of these are still in progress. The "Reference Barcode Database" (RBD) of BOLD aims to reduce the possible biases due to inadequately sampled or misidentified species. Therefore, the RBD only includes validated DNA barcodes of minimally 500 bp and only contains species represented by three or more individuals showing <2% sequence divergence [3]. The RDB currently comprises nearly 170,000 insect DNA barcodes from approximately 15,000 species. By far, the largest part of these taxa are lepidopterans (>75% of species), while the remaining 25% of insect species is distributed among 32 different orders. Although the currently barcoded taxa represent <2% of the described insect species, (thus provoking by definition a representativity problem), it is still important to have a first, general estimate of the reliability of insect DNA barcoding in relation to other methodological or conceptual issues. In order to avoid possible biases due to the large proportion of Lepidoptera in the reference database, it would be also important to separately assess the performance of DNA barcoding in different insect orders. After all, if the current DNA barcoding approaches show a low reliability in the molecular identification of insects, then the involved efforts in time, manpower and financial investments [27] could better be re-directed towards complementary or alternative identification methods. Conversely, confirming the reliability of DNA barcoding through a representative insect sample of the BOLD database would support the efforts of all research groups actively involved in insect DNA barcoding.

In this study we assess the reliability of insect DNA barcoding by performing simulations under a "best case scenario" by providing for each query one or more potential conspecific matches in the reference dataset. The objectives of this study were to (1) verify differences in the proportion of correct matches provided in different insect orders by a number of identification criteria, (2) assess if different regions of the barcode fragment are equally informative, (3) investigate relationships between barcode length and identification success, (4) evaluate how success is affected by levels of taxon coverage of the reference database.

## Results

**Figure 1:**
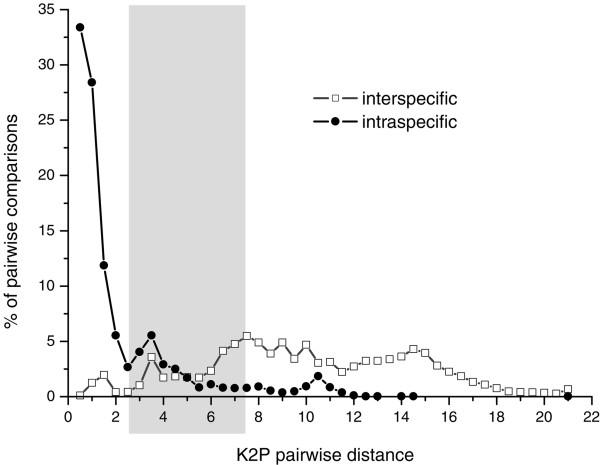
**Distance analysis**. Distributions of interspecific (white squares) and intraspecific (black circles) pairwise K2P distances resulting from the analysis of 15,948 DNA barcodes belonging to 1,995 insect species (dataset A). In grey: overlap between the 95% percentiles of intra- and interspecific distributions (2.47%<K2P < 7.64%).

The distribution of pairwise K2P distances inferred from the 15,948 DNA barcodes involving 1,995 insect species (Table 1) showed that 95% of all intraspecific distances were in the interval 0.00 - 7.64% and that 95% of mean interspecific, congeneric distances in the interval 2.47 - 21.00%. Both distance distributions were largely overlapping (Figure 1) with 27.32% of pairwise comparisons shared between the 95% percentiles of intra- and interspecific distributions (2.47%<K2P < 7.64%).

**Table 1:** Summary of DNA barcodes considered in this study.

		dataset A			dataset B	
	**n**. **barcodes**	**n**. **orders**	**n**. **species**	**n**. **barcodes**	**n**. **orders**	**n**. **species**
**Coleoptera**	628	43	114	516	16	58
**Diptera**	4,272	75	345	4,010	51	214
**Hemiptera**	901	71	164	745	39	86
**Hymenoptera**	2,067	65	160	1,961	43	107
**Lepidoptera**	7,577	495	1,167	6,567	305	662
**Orthoptera**	503	25	45	455	12	21
**total**	15,948	774	1,995	14,254	466	1,148

Dataset A (including species represented by at least two DNA barcodes) was used to describe distributions of pairwise distances, to quantify the proportion of correct matches according to Best Match (BM), Best Close Match (BCM), Neighbor-Joining Tree (NJT) and for ANOVA and regression analyses. Dataset B (including species represented by at least three DNA barcodes) was used to estimate the identification success of All Species Barcodes (ASB).

**Figure 2:**
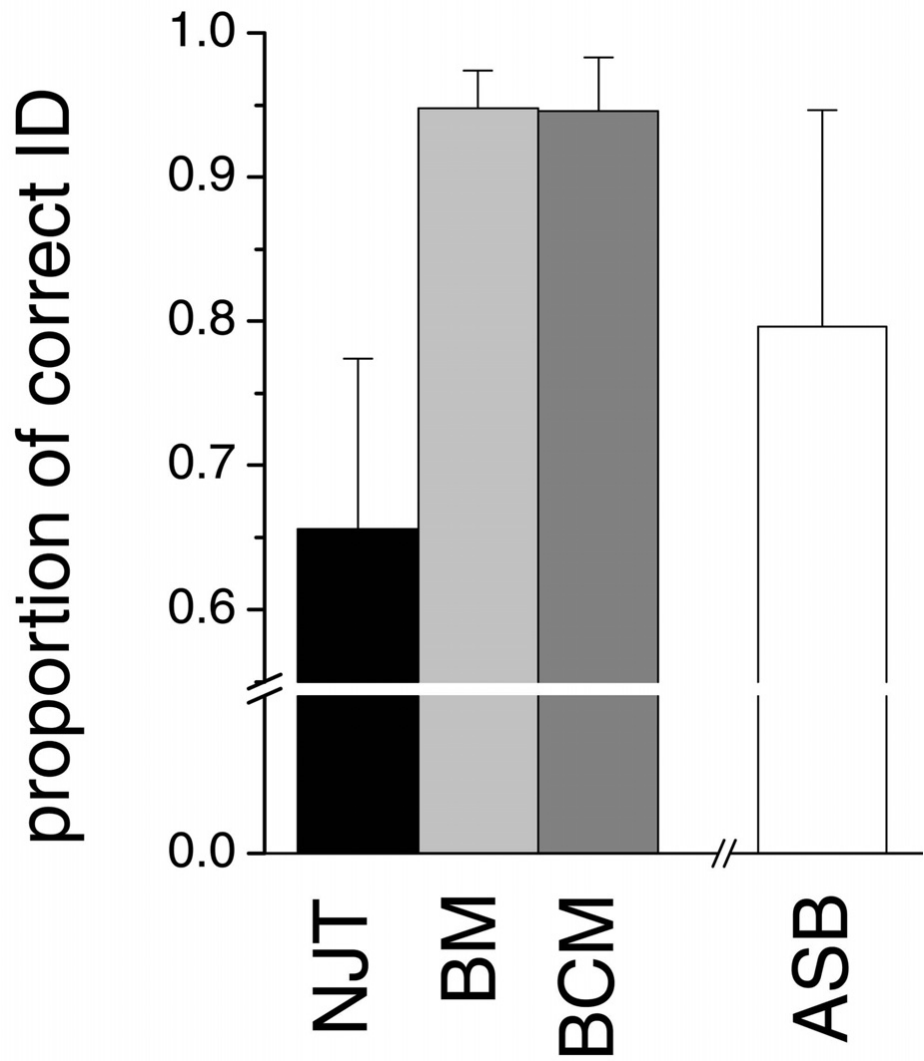
**Comparisons between identification criteria for DNA barcoding**. Proportion of correctly identified queries through Neighbor-Joining Tree (NJT), Best Match (BM), Best Close Match (BCM) and All Species Barcodes (ASB). For each identification criterion, values were averaged across six insect orders (Coleoptera, Diptera, Hemiptera, Hymenoptera, Lepidoptera, Orthoptera; SD as error bars). Dataset A (including 15,948 DNA barcodes) was used to quantify the proportion of correct identification according to BM, BCM and NJT, while dataset B (including 14,254 DNA barcodes) to quantify the identification success of ASB (see text for explanations).

The four ID criteria (Best Match: BM; Best Close Match: BCM; All Species Barcodes: ASB; and tree-based identification: NJT) yielded different proportions of correct matches (NJT = 0.656, S.D. = 0.118; BM = 0.948, S.D. = 0.026; BCM = 0.946, S.D. = 0.031; ASB = 0.796, S.D. = 0.150; Figure 2, Additional files 1, 2). ANOVA revealed highly significant differences among ID criteria and insect orders. *A posteriori *comparisons of means showed that NJT had always a significantly lower identification success than to both BM and BCM. Regardless the different numbers of taxa sampled in each order, the proportions of correct matches of BM and BCM were consistent across Coleoptera (114 species), Diptera (345 species), Hemiptera (164 species), Hymenoptera (160 species), Lepidoptera (1.167 species) and Orthoptera (45 species), while NJT showed significant variations among orders, with the highest proportion of correctly identified queries in Hymenoptera and Orthoptera and the lowest in Diptera (Table 2).

**Table 2:** ANOVA and Student-Newman-Keuls (SNK) tests.

Source of variation	*df*	MS	*F*	denominator MS for *F *ratio
Criterion = Cr	2	0.4316	34.10 ***	Cr × Or
Order = Or	5	0.0300	25.70 ***	Residual
Cr × Or	10	0.0168	19.09 ***	Residual
Residual	36	0.0009		
Total	53			
Cochran's C = 0.270, not significant				
				
**SNK test Cr:**				
BM = BCM > NJT				
				
**SNK test Cr × Or:**				
BM: Coleoptera = Diptera = Hemiptera = Hymenoptera = Lepidoptera = Orthoptera				
BCM: Coleoptera = Diptera = Hemiptera = Hymenoptera = Lepidoptera = Orthoptera				
NJT: Hymenoptera = Orthoptera > Lepidoptera > Coleoptera > Hemiptera > Diptera				

Differences in the proportion of correct matches provided by three identification criteria (BM: Best Match, BCM: Best Close Match, NJT: Neighbor-Joining Tree) were tested in six insect orders (Coleoptera, Diptera, Hemiptera, Hymenoptera, Lepidoptera, Orthoptera). Before running the analysis, the assumption of homogeneity of variances was verified using Cochran's C-test. SNK tests were used for *a posteriori *multiple comparisons of means. df: degrees of freedom, MS: mean squares estimates, *** = *p *< 0.001.

**Figure 3:**
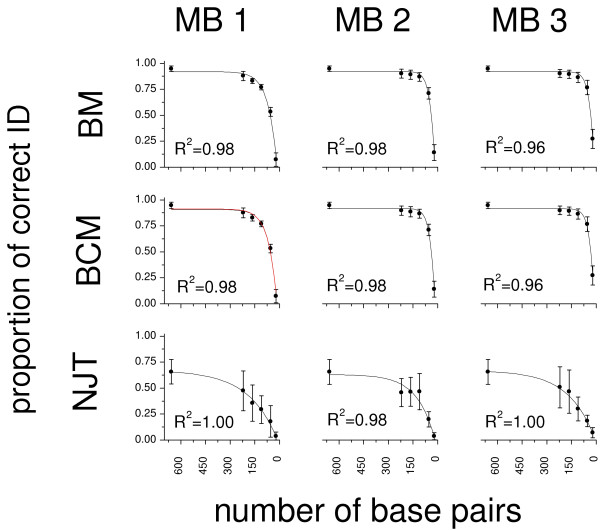
**Relationships between barcode length and identification success**. Non-linear regression of the identification success obtained by considering the full DNA barcode (550-658 bp) and three non-overlapping "mini-barcodes" (MB1: 220 bp, MB2: 219 bp, MB3: 219 bp). Each mini-barcode was reduced at both 5' and 3' ends in order to obtain fragments of 164 bp, 110 bp, 55 bp and 22 bp (corresponding to approximately to 75%, 50%, 25% and 10% of the initial mini-barcode length). For each fragment the proportion of correct identifications was calculated according to three identification criteria (BM: Best Match, BCM: Best Close Match, NJT: Neighbor-Joining Tree) and averaged across six insect orders (Coleoptera, Diptera, Hemiptera, Hymenoptera, Lepidoptera, Orthoptera, SD as error bars). Regression curves were fitted following a first order exponential decay model y = y_0 _+ ae^(-x/t)^, where y_0 _= Y offset, a = amplitude, t = exponential time constant (see Table 3).

For each identification criterion, regression and pairwise F-tests (Figure 3, Table 3 and 4) did not show significant differences in the proportion of correct matches provided by the three mini-barcodes (corresponding to the first, second and last third of the full DNA barcode). Yet, once again there were significant differences between the regression curves obtained through NJT and both BM and BCM. The proportions of correct identifications obtained from the mini-barcodes of 220 bp were relatively high when using BM (0.893, S.D. = 0.013) and BCM (0.890, S.D. = 0.013), and decreased to 50% of the value of the full barcode only when very short barcode fragments were considered (40 bp for both BM and BCM).

**Table 3:** Relationships between barcode length and identification success.

		y_0_	A	t	R^2^
	**MB1**	0.919 (0.022)	1.209 (0.124)	53.551 (6.935)	0.98
**BM**	**MB2**	0.923 (0.019)	1.745 (0.377)	26.167 (5.663)	0.98
	**MB3**	0.922 (0.019)	-1.468 (0.486)	26.444 (8.277)	0.96
					
	**MB1**	0.913 (0.025)	-1.234 (0.133)	51.135 (7.286)	0.98
**BCM**	**MB2**	0.917 (0.022)	-1.772 (0.398)	26.455 (5.716)	0.98
	**MB3**	0.915 (0.020)	-1.510 (0.525)	25.378 (8.234)	0.96
					
	**MB1**	0.671 (0.145)	-0.716 (0.138)	176.099 (107.271)	1.00
**NJT**	**MB2**	0.630 (0.116)	-0.725 (0.112)	108.431 (55.143)	0.98
	**MB3**	0.670 (0.136)	-0.687 (0.138)	160.360 (88.530)	1.00

**Figure 4:**
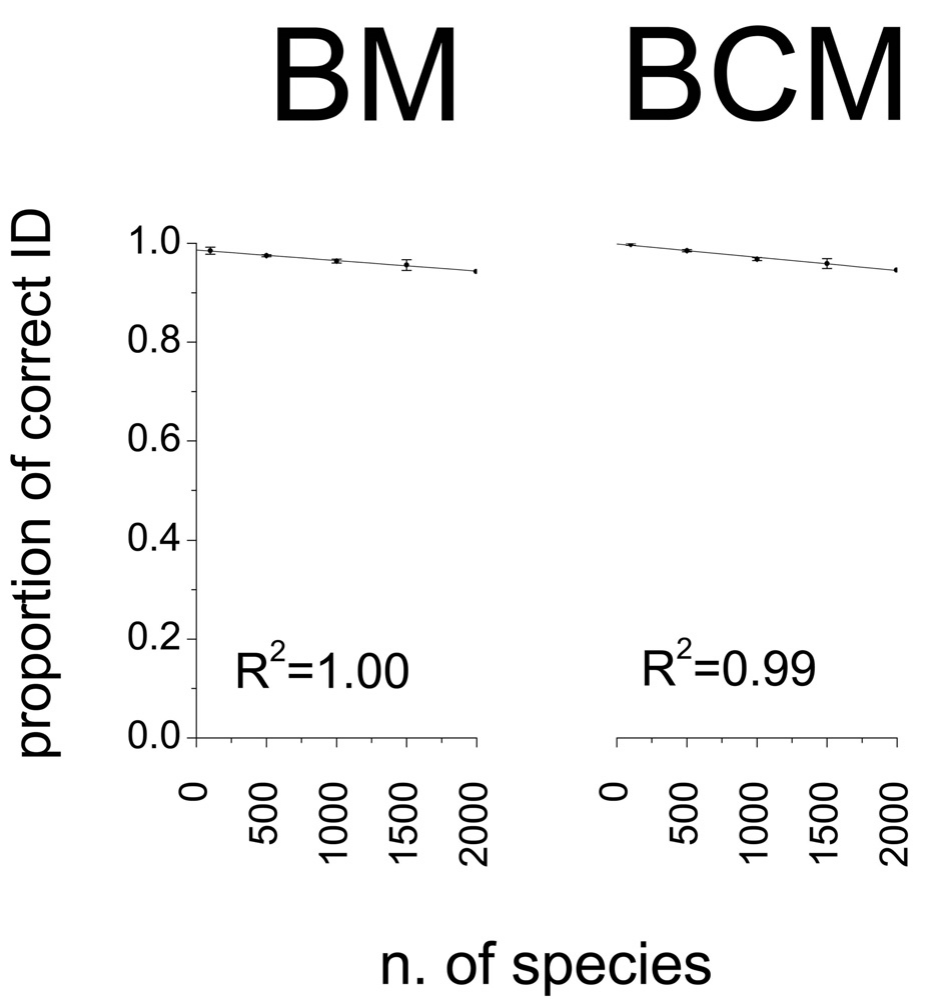
**Relationships between taxon coverage of the reference database and identification success**. Databases including 100, 500, 1,000 and 1,500 insect species were obtained by randomly sampling dataset A (three replicates for each subset of species). The identification success of Best Match (BM) and Best Close Match (BCM) was averaged across replicates (SD as error bars) and linear regression fitted after including the proportion of correct matches resulting from the analysis of the whole dataset (1,995 species).

Details of regression analyses of Figure 4. Regression curves were fitted following a first order exponential decay model y = y_0 _+ ae^(-x/t)^, where y_0 _= Y offset, a = amplitude, t = exponential time constant. The Goodness-of-fit to the non-linear model is indicated by values of R^2 ^close to 1.00.

**Table 4:** Pairwise comparisons among non-linear regression plots of Figure 2.

			BM			BCM			NJT	
		**MB1**	**MB2**	**MB3**	MB1	**MB2**	**MB3**	MB1	**MB2**	**MB3**
	**MB1**		2.348	4.307	0.001	2.307	4.423	43.972	7.433	44.186
**BM**	**MB2**	n.s.		0.831	2.476	0.003	0.864	80.475	11.491	91.195
	**MB3**	n.s.	n.s.		4.477	0.838	0.001	72.996	12.405	78.593
										
	**MB1**	n.s.	n.s.	n.s.		2.430	4.600	44.745	7.375	45.241
**BCM**	**MB2**	n.s.	n.s.	n.s.	n.s.		0.867	79.447	11.344	89.818
	**MB3**	n.s.	n.s.	n.s.	n.s.	n.s.		75.877	12.440	82.774
										
	**MB1**	**	***	***	**	***	***		0.628	1.089
**NJT**	**MB2**	*	*	*	*	*	*	n.s.		0.190
	**MB3**	**	***	***	**	***	***	n.s.	n.s.	

Above the diagonal: F-values. Below the diagonal: probability values of F-tests. n.s.: not significant, *: p < 0.05, **: p < 0.01; ***: p < 0.001. In grey: p < 0.05 after the False Discovery Rate correction.

The identification success of DNA barcoding through BM and BCM was negatively affected by the numbers of taxa included in the reference dataset, (Figure 4, Table 5), though this effect was remarkably limited as shown by the slope of the regression lines (BM: b = -2.13 × 10^-5^, BCM: b = -2.68 × 10^-5^). When passing from 100 to 1,995 species, the proportion of correct matches decreased from 0.998 to 0.948 for BM and from 0.985 to 0.946 for BCM (corresponding to -5.0% and -3.9%, respectively).

**Table 5:** Relationships between taxon coverage of the reference database and identification success.

BM		BCM	
R^2 ^= 1.00		R^2 ^= 0.99	
F = 651.91***		F = 236.178***	
a = 0.986(0.001)	t = 961.4747***	a = 0.999(0.002)	t = 465.262***
b = -2.13 × 10^-5 ^(1.74 × 10^-6^)	t = -25.53262***	b = -2.68 × 10^-5 ^(1.06 × 10^-6^)	t = -15.368***

Details of regression analyses of Figure 3. Regression lines were fitted according to the linear model y = a + bx (where a = y-intercept, b = slope). Goodness-of-fit is indicated by values of R^2 ^close to 1.00 and by the statistical significance of (1) F-test of the overall fit, (2) t-tests of individual parameters. ***: p < 0.001.

## Discussion

The relatively high proportion of correctly identified insect taxa by using two simple criteria (BM and BCM) provides a general support to the DNA barcoding method *per se*. These two distance based methods showed comparable results in terms of identification success and performed considerably better than NJT and ASB, which also showed a higher variability in the identification success of the different insect orders (Figure 2). Meier *et al*. [4] already highlighted the poorer performance of NJT, arguing that this method relies on the topology of one of all the possible Neighbor-Joining trees and does not consider the support of the nodes that separate and define species. Additionally, in case of "ties", NJT can be affected by the input order of taxa [6,28,29]. Meier also remarks that queries should be at least one node into monospecific clades, while simply clustering within a clade does not guarantee unambiguous identification [30]. Whether alternative tree reconstruction methods do better than NJT, remains to be decided. For example, Bayesian methods are currently computationally too intensive to be applied to large datasets. Nevertheless, preliminary results show that Bayesian tree reconstruction can provide considerably higher proportions of correct identifications than NJT [6]. On the other hand, the lower identification success of ASB appears related to the more stringent "decision rules" of this criterion (*i.e*. both the threshold for genetic distances and the need for all the conspecific barcodes of the query as best matches) which could lower the proportion of correct identifications.

Aliabadian *et al*. [31] stressed that the identification success of distance-based barcoding ultimately depends on the difference between intra- and interspecific divergence and in the ideal world for barcoding there is no overlap between the distributions of these two distance classes. According to Hebert *et al*. [9] the barcoding gap (*i.e*. the difference between intra- and interspecific distances) allows to assign specimens to "categories that taxonomists call species" once comprehensive reference libraries of barcodes will be available. Hebert *et al*. [9] and Aliabadian *et al*. [31] reported that in birds there is a barcoding gap, such that mean interspecific, congeneric distances are about 20-24 times larger than intraspecific ones. However, growing evidence in birds and other taxa suggests that the overlap between mean intra- and interspecific genetic distances is considerably greater when larger proportions of closely related taxa are included [13,32]. Additionally, the extent of the barcoding gap tends to be overestimated when mean intraspecific distances are used, while smallest intraspecific distances yield more consistent results [5]. Our data show that the distributions of mean intra- and interspecific congeneric distances of 1,995 insect species are largely overlapping and that the threshold Hebert *et al*. [9] proposed (mean interspecific, congeneric divergence/mean intraspecific divergence ≥ 10) is not valid to separate insect species. Surprisingly, despite there is no well-defined insect barcoding gap, distance based criteria such as BM and BCM show a remarkable identification success in our simulations. Hence, although there is a general trend for identification success to decline with increasing overlap between intra- and interspecific distances the extent of the barcoding gap should not be considered as a predictor of the identification success [5,24,30].

Different regions of the full barcode fragment provide comparable information for the molecular identification of insects. Mini-barcodes of 220 bp, though less effective than the full barcode, still yield a reasonable insect identification success (about 89% in our simulations). Though less encouraging than previous simulations based on insect COI fragments of comparable lengths [25,26], our results suggest that mini-barcodes could indeed represent a cost-effective way of building reference databases for the molecular identification of species. However, it would be important to carefully consider the trade offs between mini-barcode length and proportions of correctly identified insect taxa and clearly establish thresholds for the minimal lengths and features of the mini-barcodes that will be incorporated into reference databases.

## Conclusions

Although, our simulations show that the probability of false identifications of queries with conspecifics in the reference database is relatively limited (up to 5.2% when using BM), there remains a logical problem with insect DNA barcoding due to the limited proportion of referenced taxa in BOLD and RBD. Indeed, in a real life situation, one does not know in advance whether or not a query is represented in BOLD [4], but one can reasonably assume that there is a good chance that it belongs to the 98% of insect species that still have to be barcoded. According to BM (*i.e*. the identification criterion currently adopted in BOLD) all queries not represented in the reference databases will be, in the best case, assigned to the most closely related taxon available in the database, thus generating a considerable amount of false identifications. Accordingly, whenever the null hypothesis H_0_: Q_x _= Sp_A _(where Q_x _= a query about unknown species X and Sp_A _= species A) is accepted, then this may imply either (1) a correct identification of X as species A or (2) a Type II error (H_0 _= accepted when false), where species X is wrongly identified as A because it is not represented in the reference database and hence is associated with another species. Conversely, if H_0 _is rejected (i.e. species X is not A), then this may imply either (1) a correct decision that species X is indeed not A or (2) a Type I error (H_0 _= rejected when true), where X is erroneously not recognized as being A. The proportion of Type II errors is hardly predictable, but we should expect higher levels of Type II errors in reference database with poor taxon coverage. Additionally, when using the reference database of BOLD, only part of the queries without conspecifics in the database are misidentified, as the identification procedure is aborted whenever the genetic divergence between query and best match exceeds an arbitrary threshold of 3% [3]. The simulations performed in this study allowed to quantify the proportion of Type I errors in a large database of insect DNA barcodes. We observed a trend toward a progressive increase of false identifications in databases with larger taxon coverage. When passing from 100 to 1,995 species, the proportion of Type I errors increases of 5.0% for BM (from 0.2% to 5.2%) and of 3.9% for BCM (from 1.5% to 5.4%). So, the proportion of correct identifications and the number of species in the reference database are inversely related, even if the slope of the linear regression is close to zero. These results suggest that the combined effects of Type I and II errors can heavily affect the reliability of DNA barcoding in the molecular identification of insects. However, biases related to Type II errors and the limited taxon coverage of the currently available databases could be circumvented by adopting a "negative" approach, *i.e*. by using DNA barcoding to verify the lack of correspondence between an unknown query and a list of well-known referenced target taxa. This approach would allow ruling out Type II errors and performing a "negative identification" (H_0 _rejected), which is only subject to Type I errors. Given that levels of genetic variability of a group of target species are adequately represented in the reference database, DNA barcoding could hence still be profitably used in quarantine interception of insects of economical importance by indicating that a sample of interest does not include a list of referenced pests. Species of economical significance are generally better characterised from a molecular perspective and are among the best represented in reference databases. Cameron *et al*. [27] argued that the relatively small number of species that need to be identified for quarantine purposes makes the assembly of a high quality reference database much easier than for all of life and suggested it would be more practical to use barcoding as an entirely DNA based quarantine system. However, it is of crucial importance to understand how the proportion of Type I and Type II errors will change once hundreds of thousands of insect taxa will be included in the reference databases. The lower identification success observed in this study for larger databases should be considered only as a very preliminary indication as the simulations could only include a limited fraction of the global taxonomic diversity of insects (from 100 to 1,995 species). Hence, a major challenge for insect DNA barcoding will be to understand if the error levels associated to the currently adopted distance-based identification criteria will still be acceptable once reference databases will have a more representative coverage of insect taxonomic diversity.

## Methods

We used the DNA barcodes available in June 2009 in BOLD http://www.barcodinglife.org and downloaded all the publicly available sequences belonging to the orders Coleoptera, Diptera, Hemiptera, Hymenoptera, Lepidoptera and Orthoptera. The dataset was extended with 324 Tephritid (Insecta: Diptera) DNA barcodes collected by the Royal Museum for Central Africa in the framework of the Tephritid Barcoding Initiative, a demonstrator project, initiated by CBOL. DNA barcodes were trimmed in order to include only the barcode region, namely the 658 bp COI fragment amplified by the "universal primers" of Folmer *et al*. [1]. Sequences shorter than 550 bp and DNA barcodes with incomplete species information (*e.g*. sequence names including *sp*., *cf*., *nr*. etc.) were discarded. Nucleotide sequences were aligned using the default parameters of the IUB scoring matrix of ClustalW, as implemented in Bioedit 7.0 [33]. Sequences were aligned in blocks of 800 and all the blocks were aligned to a single, haphazardly chosen, profile sequence (accession GQ154187). The blocks were visually inspected to check for possible incongruence (*i.e*. the occurrence of gaps which were not multiples of 3 bp). Each block was then pasted into the data matrix until all sequences were aligned. In order to allow comparisons between our results and large part of the current literature on DNA barcoding, we used pairwise Kimura's two parameter (K2P) distances [34] and plotted the frequency distributions of intraspecific and mean interspecific, congeneric distances.

We estimated the proportion of correct matches provided by the four identification criteria described by Meier *et al*. [4]. These included three distance-based criteria (Best Match: BM; Best Close Match: BCM; All Species Barcodes: ASB) and the tree-based identification (tree-identification: NJT). According to BM, each query was assigned the species name of its best-matching barcode regardless of how similar the query and barcode sequences were. In our simulation, identification was considered successful when both sequences were from the same species. BCM relies on a threshold value of sequence similarity. The threshold was estimated from dataset A (see below) by establishing a frequency distribution of pairwise intraspecific distances and determining the distance below which 95% of all intraspecific distances are found. BCM first identified the best barcode match of a query and then assigned the species name of that barcode only if the distance between query and barcode was below the threshold. The simulation of ASB proceeded as for BM but assigned a species name only when all the conspecifics of the query topped the list of best matches. NJT considered an identification to be correct if the query and all its conspecific sequences formed a monospecific clade. We evaluated the identification success (*i.e*. the proportion of correct matches) of the four criteria by using two separate datasets (Table 1): (1) species of which a query would find at least one conspecific match in the reference database (dataset A, 15,948 DNA barcodes, 1,995 species, Additional file 3) and (2) species of which a query would find at least two conspecific matches in the reference database (dataset B, 14,254 DNA barcodes, 1,148 species, Additional file 4). Dataset A was used to evaluate the proportion of correct matches according to BM, BCM and NJT, while dataset B to evaluate the identification success of ASB. SpeciesIdentifier1.5 [4] was used to calculate pairwise K2P distances and to quantify the proportion of correct matches according to BM, BCM and ASB. The identification success of NJT was quantified using PAUP* [35] to reconstruct a Neighbor-Joining tree (K2P distance, ties randomly broken) and calculating the NJT identification success by using a Perl script developed for this purpose (TreeCode.v1, Additional file 5).

Differences in the identification success of (1) BM, BCM and NJT and (2) Coleoptera, Diptera, Hemiptera, Hymenoptera, Lepidoptera, Orthoptera were tested by a 2-way model of analysis of variance (ANOVA). ANOVA was based on three independent replicates, *sensu *Underwood [36], which were obtained by (1) randomly assigning the DNA barcodes of each order to three groups (each corresponding to an identification criterion) and (2) dividing each group in three sub-groups. Each replicate was represented by the proportion of correct matches calculated in each sub-group, according to the identification criterion of each group. Identification criterion (Cr, 3 levels, fixed) and insect order (Or, 6 levels, random) were considered as orthogonal variables. The assumption of homogeneity of variances was verified through Cochran's C-test. Student-Newman-Keuls (SNK) tests were used for *a posteriori *multiple comparisons of means [36]. The identification success of ASB (calculated from dataset B) was qualitatively compared with the proportion of correct matches obtained for BM, BCM and NJT (calculated from dataset A).

Relationships between barcode length and identification success were analysed through non-linear regression. The DNA barcodes of dataset A were divided in three non-overlapping "mini-barcodes" of 220, 219 and 219 bp corresponding to the first, second and last third of the barcode region (hereafter MB1, MB2, MB3). The number of bp of each mini-barcode was further reduced at both 5' and 3' ends in order to obtain fragments of approximately 75%, 50%, 25% and 10% of the initial mini-barcode length (164 bp, 110 bp, 55 bp and 22 bp, respectively). For each fragment the proportion of correct identifications was calculated according to three identification criteria (BM, BCM, NJT) and averaged across the six insect orders (Coleoptera, Diptera, Hemiptera, Hymenoptera, Lepidoptera, Orthoptera). Non-linear regression fitting was implemented for each combination of identification criterion and barcode fragment following the first order exponential decay model y = y_0 _+ ae^(-x/t)^, where y_0 _= Y offset, a = amplitude, t = exponential time constant [37]. Differences among regression curves were verified through pairwise F-tests. Probability values of repeated comparisons were corrected for Type I errors using the False Discovery Rate procedure [38]. Relationships between levels of taxon coverage in the reference database and identification success were investigated through linear regression. The DNA barcodes of dataset A were randomly sampled in order to obtain reference databases including subsets of 100, 500, 1000 and 1,500 insect species. Three independent replicates were obtained for each subset. The identification success of BM and BCM was averaged across the three replicates of each subset and regressions were fitted after including the proportion of correct matches resulting from the analysis of the whole dataset (1,995 species). Regression analyses were implemented in OriginPro v7 http://www.originlab.com after comparing the Goodness-of-fit of alternative models.

## Authors' contributions

MV conceived the study, performed the statistical analyses and drafted the manuscript. BN developed the bioinformatic tools and participated to the analysis of data and manuscript preparation. TB and MDM contributed to the study design and critically reviewed the manuscript. All authors read and approved the final version of the manuscript.

## Supplementary Material

Additional file 1 **Summary of simulations on Dataset A**. Click here for file

Additional file 2 Summary of simulations on Dataset B. Click here for file

Additional file 3 **Dataset A**. Accession numbers and alignment of 15,948 DNA barcodes (>550 bp) belonging to 1,995 insect species, each represented by at least two sequences. Click here for file

Additional file 4 **Dataset B**. Accession numbers and alignment of 14,254 DNA barcodes (>550 bp) belonging to 1,148 insect species, each represented by at least three sequences. Click here for file

Additional file 5 **TreeCode.1.0**. Perl script developed to quantify the proportion of correctly identified queries according to the NJT criterion. Click here for file
